# The complete mitochondrial genome of a marine triclad *Miroplana shenzhensis* (Platyhelminthes, Tricladida, Maricola)

**DOI:** 10.1080/23802359.2022.2079102

**Published:** 2022-06-02

**Authors:** Jia-Jie Huang, Yuan-Yuan Liao, Wei-Xuan Li, Jun-Yu Li, An-Tai Wang, Yu Zhang

**Affiliations:** a Shenzhen Key Laboratory of Marine Bioresource and Eco-environmental Science, College of Life Sciences and Oceanography, Shenzhen University, Shenzhen, Guangdong, P. R. China; b State Key Laboratory of Protein and Plant Gene Research, Center for Bioinformatics, School of Life Sciences, Peking University, Beijing, P. R. China; c Guangdong Engineering Research Center for Marine Algal Biotechnology, College of Life Sciences and Oceanography, Shenzhen University, Shenzhen, Guangdong, P. R. China

**Keywords:** Mitogenome, gene order, molecular phylogeny

## Abstract

The complete mitochondrial genome (mitogenome) of *Miroplana shenzhensis* Yu & Wang, 2013 is reported in the present study, representing the second mitogenome recorded in the suborder Maricola. The circular mitogenome is 14,344 bp in length, containing 12 protein-coding genes, 2 ribosomal RNAs and 22 transfer RNAs. Comparative analysis on mitochondrial gene order reveals a rearrangement in the suborder Maricola, indicating that mitochondrial gene order is conserved only in Continenticola, and is divergent across Tricladida. Phylogenetic analysis shows *M. shenzhensis* is clustered with an another marine triclad, forming a well-supported monophyletic group of Maricloan.

*Miroplana shenzhensis* Yu & Wang, 2013 was classified into the genus *Miroplana* (Platyhelminthes, Tricladida, Maricola) according to its morphological features (Yu et al. 2013), while a more recent 18S and 28S rDNA based phylogenetic study demonstrated the phylogenetic position of the genus *Miroplana* (Li et al. 2019). Intriguingly, *Miroplana* exhibits good adaptation to both brackish water and freshwater habitats, which is similar to *Sluysia triapertura* (Souza et al. 2018) and species of the genus *Paucumara* (Li et al. 2021, Chen et al. 2019, Sluys 1989) and *Pentacoelum* (Sluys et al. 2015), but stands out of the majority of suborder Maricola species. Therefore, it will be important to further resolve the phylogenetic position of *M. shenzhensis* among triclad species using molecular markers other than 18S and 28S rDNA. However, complete mitogenome is only available for one marine triclad, namely *Obrimoposthia wandeli* (Yang et al. 2019). In this study, we present the mitogenome of *M. shenzhensis*, representing the second mitogenome belonging to the suborder Maricola.

The specimen of *M. shenzhensis* was collected on 20 May, 2018 from mangrove wetlands of Shenzhen Waterfront Ecological Park, Guangdong, China (22°52.27′N, 114°00.34′E). The genomic DNA was extracted by Li et al (2019) and currently deposited at College of Life Sciences and Oceanography, Shenzhen University (Zhang Yu, biozy@szu.edu.cn) under the voucher number SW001. We used the REPLI-g Midi Kit (QIAGEN, Hilden, Germany) to amplify the genomic DNA. Paired-end sequencing was conducted on the Illumina Hiseq 2500 platform (Novogene, Beijing, China). The mitogenome sequences were assembled using MitoFlex v0.2.9 (Li et al. 2021). MITOS web server was used for gene annotation (Bernt et al. 2013), while the start and stop codons and the functional regions of the genes were verified by BLASTN (Altschul et al. 1997) with the query sequences from the closely related species *O. wandeli* (Yang et al. 2019). Multiple sequences alignment (MSA) was performed using MACSE v2.03 (Ranwez et al. 2018). MSAs were subsequently trimmed using Gblocks v0.91b (Talavera and Castresana 2007). Substitution saturation test (Xia et al. 2003; Xia and Lemey 2009) for each protein-coding gene (PCG) was carried out in DAMBE6 (Xia 2017), while the third positions of all PCGs were excluded for downstream analysis due to nucleotide substitution saturation. Best-fit evolution model for each PCG was selected by PartitionFinder2 (Lanfear et al. 2017). The phylogenetic trees were constructed by Maximum Likelihood (ML) and Bayesian Inference (BI) methods, respectively. For ML, standard bootstrap analysis with 10,000 replications was performed by IQ-TREE v2.1.2 (Chernomor et al. 2016; Minh et al. 2020). While for BI, MrBayes v3.2.6 (Ronquist et al. 2012) was applied with 5,000,000 generations, sampling every 5,000 generations.

The circular mitogenome of *M. shenzhensis* is 14,344 bp in length and contains 12 PCGs, two rRNAs and 22 tRNAs. The nucleotide base composition is 28.7% A, 10.8% C, 15.8% G, and 44.7% T, with a total A + T content of 73.4%. *ATP8* gene, through automatic annotation, was noted as missing in the mitogenome of *M. shenzhensis*. *rrnL* is situated at 5′ upstream of *rrnS* in the mitogenome of *M. shenzhensis* and *O. wandeli*, which is opposite to the unique arrangement of ribosomal genes in the suborder Continenticola (Solà et al. 2015). Unexpectedly, except a few gene groups, namely *ND4-ND4L* and *ND3-ND2*, which are conserved in their arrangement in mitogenome, the PCG arrangement of *M. shenzhensis* differs from those of other known triclads. The current data show that the gene order is highly conserved across the suborder Continenticola while being divergent not only within Maricola, but also between Continenticola and Maricola. As such, our data provides a valuable addition to the previous perspective that gene order is strikingly conserved among Tricladida (cf. Ross et al. 2016).

The phylogeny was inferred from 12 concatenated PCG sequences of *M. shenzhensis* and 12 species of Tricladida, while two species of Polycladida were included as outgroups. The phylogenetic trees obtained from both BI and ML analysis share identical topologies, and being well-supported in most nodes (Figure 1). *M. shenzhensis* is more closely related to a marine triclad *O. wandeli* than other freshwater and terrestrial Continenticolans, forming a monophyletic group consists of Maricolans with high supporting values.

**Figure 1.**
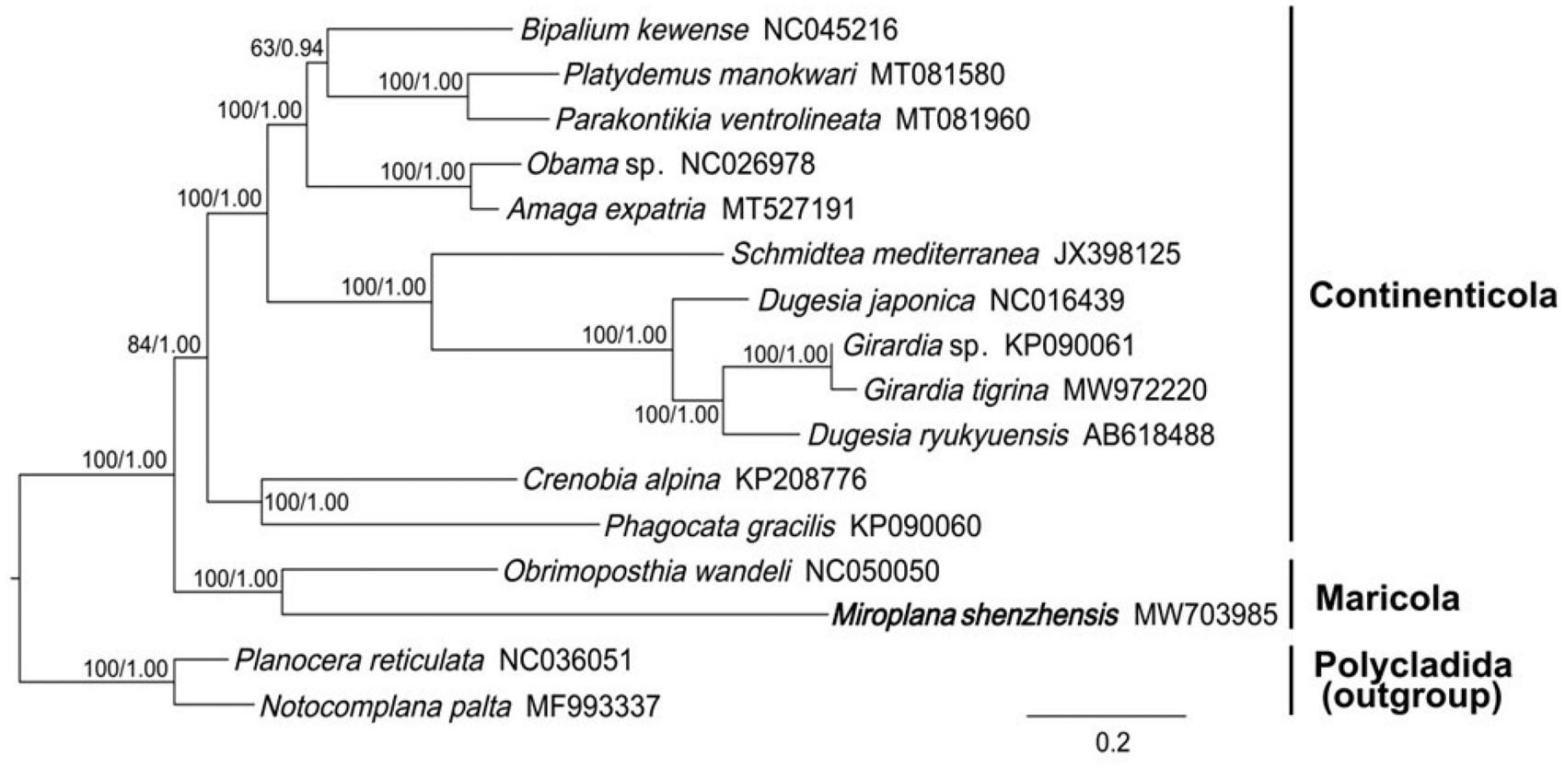
Bayesian inference phylogenetic tree topology inferred from the concatenated sequences of 12 PCGs. Nodal values indicate support values (bootstrap/posterior probability).

In conclusion, our study provides baseline information for future research regarding the origin and evolution in the suborder Maricola, suggesting the possible existence of the divergence of mitochondrial gene order in Tricladida. To better understand the phylogenetic relationships among species of Tricladida, it will be important to expand the mitogenome analysis within the order.

## Data Availability

The genome sequence data that support the findings of this study are openly available in Genbank of NCBI at https://www.ncbi.nih.gov under the accession no. MW703985. The associated BioProject, SRA, and Bio-Sample number are PRJNA777831, SRR16770661, and SAMN22883523 respectively.
